# Advances in CAR-NK cell therapy for hematological malignancies

**DOI:** 10.3389/fimmu.2024.1414264

**Published:** 2024-06-28

**Authors:** Rui Yang, Yun Yang, Rui Liu, Yiwen Wang, Ruoyu Yang, Aili He

**Affiliations:** ^1^ Department of Hematology, The Second Affiliated Hospital of Xi’an Jiaotong University, Xi’an, Shaanxi, China; ^2^ Xi’an Key Laboratory of Hematological Diseases, Xi’an, Shaanxi, China

**Keywords:** chimeric antigen receptor, immunotherapy, NK cells, CAR-NK, hematological malignancies

## Abstract

Chimeric antigen receptor T (CAR-T) cell therapy has revolutionized the treatment of hematological malignancies, demonstrably improving patient outcomes and prognosis. However, its application has introduced new challenges, such as safety concerns, off-target toxicities, and significant costs. Natural killer (NK) cells are crucial components of the innate immune system, capable of eliminating tumor cells without prior exposure to specific antigens or pre-activation. This inherent advantage complements the limitations of T cells, making CAR-NK cell therapy a promising avenue for hematological tumor immunotherapy. In recent years, preclinical and clinical studies have yielded preliminary evidence supporting the safety and efficacy of CAR-NK cell therapy in hematological malignancies, paving the way for future advancements in immunotherapy. This review aims to succinctly discuss the characteristics, significant therapeutic progress, and potential challenges associated with CAR-NK cell therapy.

## 1 Introduction

Chimeric antigen receptor T (CAR-T) cell therapy has emerged as a major breakthrough in cancer treatment in recent years. Notably, CARs targeting B-lineage-specific antigens, such as CD19 and CD20, have shown particular success (1). As of today, the Food and Drug Administration (FDA) has approved six CAR-T cell therapies: four CD19-directed T-cell products (tisagenlecleucel/KYMRIAH, axicabtagene ciloleucel/YESCARTA, brexucabtagene autoleucel/TECARTUS, and lisocabtagene maraleucel/BREYANZI) and two B-cell maturation antigen (BCMA)-targeted T-cell products (idecabtagene vicleucel/ABECMA and ciltacabtagene autoleucel/CARVYKTI). This approach has demonstrably improved outcomes for patients with recurrent/refractory hematological malignancies. However, significant challenges remain in the application of CAR-T cell therapy to solid tumors. These challenges include the scarcity of tumor-specific antigens and the limited infiltration of immune cells into solid tumors (2, 3). Despite its remarkable achievements, CAR-T cell therapy also faces inherent complexities and safety considerations associated with autologous T-cell therapy. These limitations, including the cytokine release syndrome (CRS) and the immune effector cell-associated neurotoxicity syndrome (ICANS), necessitate the exploration of alternative therapies (4, 5). These limitations have spurred interest in alternative therapies such as natural killer (NK) cell therapy.

## 2 Biological characteristics of NK cells

NK cells, which are derived from CD34^+^ lymphoid progenitor cells in the bone marrow, account for approximately 15% of the lymphocyte population and serve as important components of the innate immune defense system (6–9). Functionally similar to CD8^+^ cytotoxic T cells, NK cells lack antigen-specific receptors. This enables them to recognize and eliminate abnormal cells in an antigen-independent manner, distinguishing them from T cells within the lymphocyte lineage (10, 11). Circulating NK cells can be further categorized into two major subsets based on the surface expression density of CD56 (neural cell adhesion molecule) and CD16 (6): immature CD56^bright^CD16^−/low^ NK cells and mature CD56^dim^CD16^+^ NK cells. The highly proliferative immature subset plays a critical role in immunoregulation through the production of cytokines, particularly interferon gamma (IFN-γ) (11). Upon cytokine stimulation, CD56^bright^CD16^−/low^ NK cells can differentiate into cytotoxic CD56^dim^CD16^+^ NK cells (9). Notably, mature NK cells, constituting approximately 90% of peripheral blood NK cells, exhibit potent cytotoxic activity (12, 13).

NK cells distinguish abnormal cells from normal cells through a delicate interplay between the activating and inhibitory surface receptors. The functional state of an NK cell is ultimately determined by the balance of the signals received from these receptors (10, 14). The activating receptors encompass a diverse group, including partial cytokine-binding receptors (e.g., interleukin-2 receptor), natural cytotoxicity receptors (NCRs) such as NKp44, NKp30, and CD16, and others such as the SLAM family receptors and the activated forms of killer cell immunoglobulin-like receptors (KIRs) (11, 15). NKG2D is a typical activating receptor whose ligands are only generally expressed on the surface of abnormal cells such as tumor or stressed cells and can activate the ability of NK cells to kill abnormal cells upon binding to the NKG2D ligands (NKG2DL) (16). This property makes NKG2D an attractive target for cancer immunotherapy (17). Inhibitory receptors fall into two major categories: those recognizing the major histocompatibility complex class I (MHC-I) molecules and those that do not. MHC-I-specific inhibitory receptors include NKG2A, inhibitory KIRs (e.g., KIR2DL1/2/3 and KIR3DL1/2), and LLT1. PD-1 and 2B4 are examples of the non-MHC-I-specific inhibitory receptors (10, 11).

**Figure 1:**
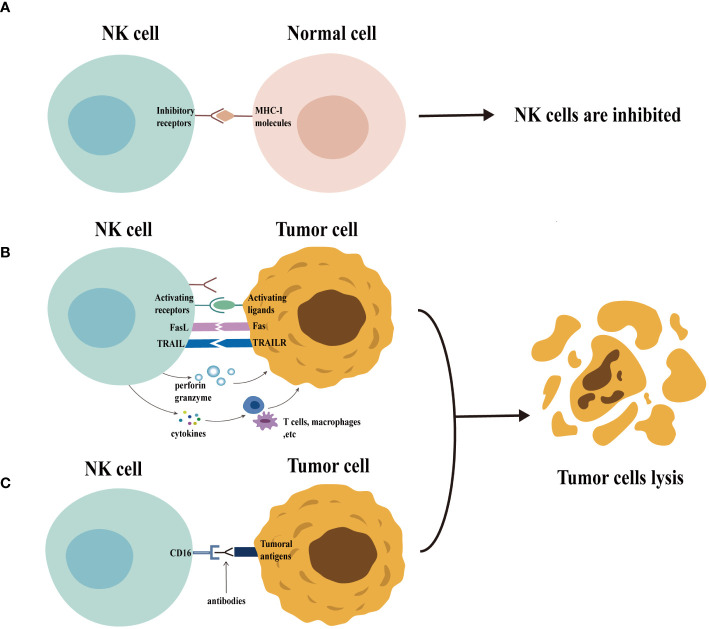
Function of natural killer (NK) cells. Normal cells express MHC-I molecules on their surface. When the inhibitory receptors on NK cells bind to these molecules, NK cells are inhibited and are unable to exert their cytotoxic potential (panel **A**) . In contrast (panel **B**), the expression of MHC-I molecules is usually downregulated in abnormal cells (e.g., tumor cells), accompanied by the expression of a variety of activating ligands, which leads to the activation of NK cells. The activated NK cells exert their cytotoxic potential by secreting granules containing perforin and granzyme and by the death receptor pathway. The activated NK cells can also secrete cytokines that regulate the function of other immune cells, including T cells and macrophages, thus enhancing the immune response. Furthermore, NK cells can be activated through CD16 recognition of target cell-binding antibodies, enabling them to exert their cytotoxic potential through ADCC effects (panel **C**).

NK cells employ a multifaceted arsenal to eliminate target cells (**Figure 1**). Activated NK cells degranulate, releasing cytolytic granules containing perforin and granzymes. Perforin creates pores in the target cell membrane, allowing granzymes to enter and induce cell death (apoptosis) (18, 19). In addition, the activated NK cells upregulate death ligands, such as the Fas ligand (FasL) and the tumor necrosis factor (TNF)-related apoptosis-inducing ligand (TRAIL), on their surface. These ligands can bind to the death receptors on target cells, triggering apoptosis in a process known as the death receptor pathway (18, 20). Mature NK cells, characterized by a high CD16 expression, can also mediate antibody-dependent cellular cytotoxicity (ADCC). During this process, CD16 binds to the Fc portion of the antibodies already bound to the target cells, leading to target cell lysis (21). Finally, the activated NK cells secrete various cytokines that influence the immune response (22).

## 3 Construction of CAR-NK

**Figure 2:**
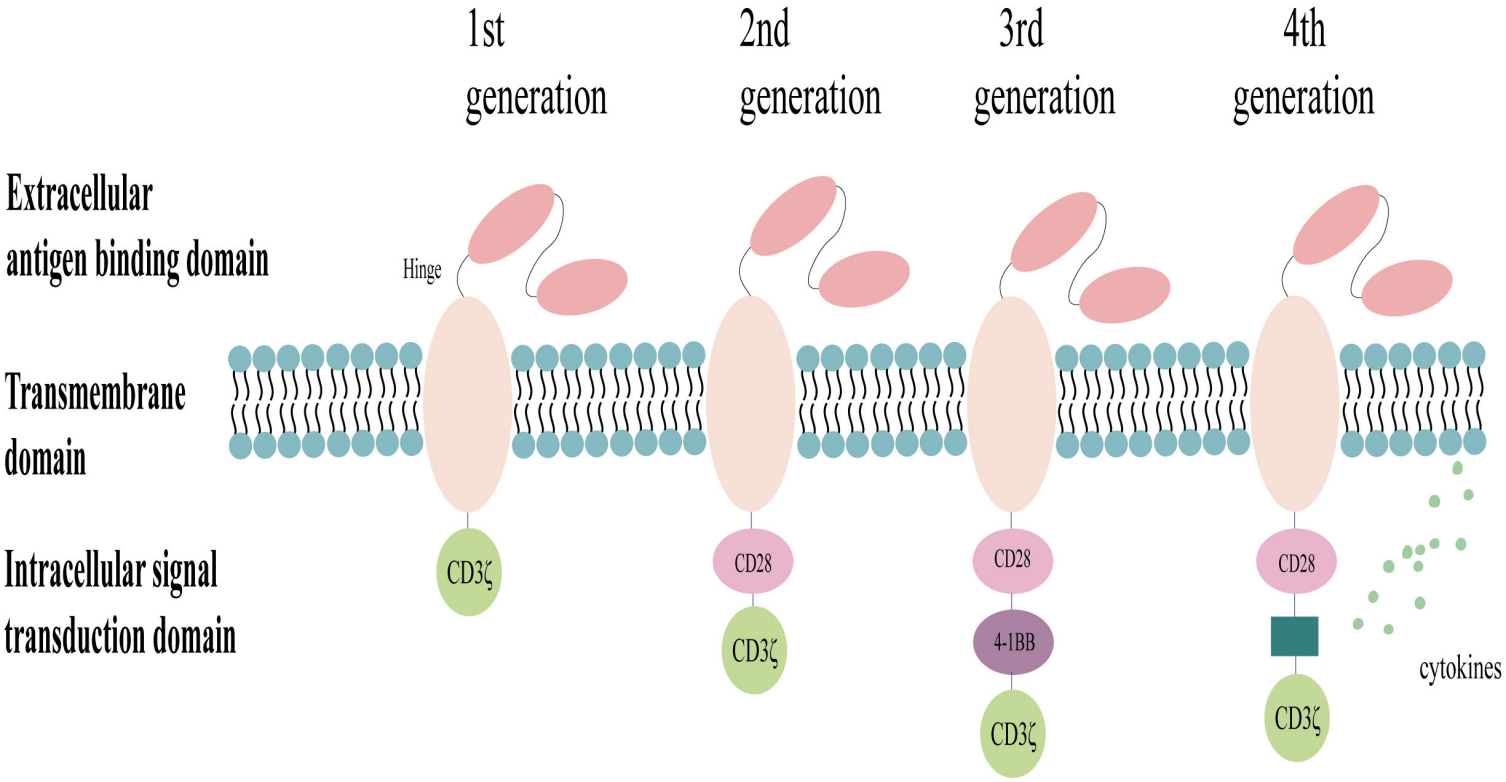
Structure of the chimeric antigen receptor (CAR). The design of CARs has evolved through generations, with the first generation incorporating a single CD3ζ signaling domain for transient activation, the second generation adding a co-stimulatory molecule (CD28 or 4–1BB) for enhanced signaling, the third generation incorporating two co-stimulatory molecules for further signal transduction efficiency, and the fourth generation co-expressing cytokines or suicide genes to improve targeting, efficacy, and the safety profiles.

Chimeric antigen receptors (CARs) are engineered proteins composed of several key domains: extracellular antigen-binding domains for recognizing tumor-associated antigens (TAAs), flexible hinge regions, transmembrane domains anchoring the CAR to the cell membrane, and intracellular signaling domains that directly activate immune cells to eliminate the target cells (3, 23). The design of CARs has become increasingly sophisticated with advancements in research (**Figure 2**). The current exploration focuses on fifth-generation CARs, which may incorporate features such as inducible cytokine production through the inclusion of transcription factor binding sites. For instance, the integration of an interleukin-2 (IL-2) Rβ signaling domain, which activates STAT3, has been proposed to enhance T-cell activation and proliferation via both the CD3ζ/CD28 and JAK-STAT 3/5 pathways (24). Notably, most CAR-NK cell studies currently utilize first- or second-generation CAR structures adapted from CAR-T cell designs (23, 25). Research efforts are ongoing to improve the efficacy of CARs for targeted antitumor therapy. These efforts include optimizing the signaling domains, developing dual/multi-targeted CARs, and selecting less immunogenic extracellular fragments (26, 27). A recent study exploring a novel combination of intracellular signaling domains (CD3ζ-2B4) has demonstrated significant functional improvements in CAR-NK cells *in vitro* (28).

NK cells can be derived from various sources (29), including peripheral blood (PB), umbilical cord blood (UCB), established NK cell lines (particularly the NK-92 cell line), and human pluripotent stem cells (hPSCs). This wider range of potential sources makes NK cells more readily available compared with T cells, with each source offering distinct characteristics, detailed in **Table 1**.

**Table 1:** Characteristics of natural killer (NK) cells from different sources.

Source	Advantages	Disadvantages
**PB-NK**	Mature phenotype (30) Excellent killing capability (23)	Poor amount (31) Limited proliferation (30)
**UCB-NK**	Enhanced proliferation (32) Reassuring security (33)	Less mature (34)
**hPSCs**	Rich sources (35) Low immune risk (36)	Uncertain safety (37) Complex differentiation (33)
**NK-92 cell line**	Genetic modification (38) Nice accessibility (39)	Tumorigenicity risk (40) Lack expression of CD16 (41)

PB, peripheral blood; UCB, umbilical cord blood; hPSC, human pluripotent stem cells.

Currently, two main approaches are used to introduce genetic material into NK cells: viral transduction (including lentiviral and retroviral vectors) and non-viral transduction (encompassing mRNA electroporation and transposon systems). Of these, lentiviral vectors represent a classic and widely employed method. However, their lower efficiency in transducing NK cells can limit the application of CAR-NK therapy. Strategies to improve the lentiviral transduction efficiency, such as employing multiple transduction rounds or co-applying transduction enhancers, have been detailed in previous reviews (35, 42). A summary of the key characteristics of the different transduction systems is in **Table 2**.

**Table 2:** Characteristics of different transduction systems.

Method		Characteristics	References
**Viral transduction**	Retroviral vectors	Long-term expression in dividing cells, insertional mutagenesis, and immune response	(25, 33, 43, 44)
	Lentiviral vectors	Lower immunogenicity and lower risk of insertional mutagenesis than retrovirus, non-cell cycle dependent	(25, 45–47)
**Non-viral transduction**	mRNA electroporation	High efficacy, low cost, low risk of insertional mutagenesis and immune response, transient expression, risk of cell death	(43, 46, 47)
	Transposon	Moderate transduction rate, stable expression and low cost, risk of insertional mutagenesis	(45, 47)

## 4 Potential advantages of CAR-NK

At present, CAR-T cell therapy is expensive and time-consuming. The use of autologous T cells minimizes the risk of graft-*versus*-host disease (GvHD), a serious complication in allogeneic cell therapy (48, 49). However, this reliance on autologous T cells presents logistical challenges for large-scale clinical application due to limitations in T-cell collection and manipulation (50). NK cells, unlike CAR-T cells, pose a significantly lower risk of GvHD (51) and are abundantly available. This inherent accessibility presents a distinct advantage for NK-based therapies. In addition, the high costs associated with the currently approved CAR-T products, such as the Kymriah with a list price of $475,000, limit patient access (52). The study by Jagannath et al. (53) further highlighted the financial burden of CAR-T therapy, reporting pre- and peri-infusion healthcare costs exceeding US $15,000 for relapsed/refractory multiple myeloma (R/RMM) patients alone. Indeed, these high costs create a barrier for many patients and necessitate the exploration of alternative treatment options.

Despite promising response rates, CAR-T cell therapy carries a significant risk of CRS and ICANS, a concern that cannot be disregarded during clinical application (54). In contrast, preliminary clinical trials suggest a potentially safer profile for CAR-NK cell therapy (55). This difference may be attributed to the distinct cytokine profiles released by the activated CAR-T and CAR-NK cells (3). Upon activation, CAR-T cells release a surge of inflammatory cytokines, such as IL-2, IL-6, and tumor necrosis factor alpha (TNF-α), which are known contributors to CRS and ICANS (54). Conversely, the activated CAR-NK cells primarily produce IFN-γ and granulocyte–macrophage colony-stimulating factor (GM-CSF) (56), cytokines that lack a demonstrated role in the pathogenesis of CRS and ICANS (54). Supporting this notion, a clinical trial using cord blood-derived CAR-NK cells for B-cell malignancies observed minimal elevation of CRS-associated inflammatory factors such as IL-6 and TNF-α, suggesting a potentially safer therapeutic approach (55).

It is now understood that CAR-T cells recognize TAAs through their CARs. This recognition appears to require a higher TAA density compared with the activation of T cells via their T-cell receptors (TCRs) (50, 57, 58). Consequently, antigen escape, where tumor cells downregulate or lose TAA expression, is a frequent challenge in CAR-T cell therapy, limiting its *in vivo* efficacy. In contrast, CAR-NK cells exhibit a dual killing capacity: CAR-dependent and CAR-independent pathways. The latter pathway leverages the inherent cytotoxicity of NK cells, allowing them to eliminate tumor cells even with partial or complete TAA loss (59). This theoretical advantage suggests a potentially high antitumor potential for CAR-NK cells. However, the limited research available directly compares the antitumor efficacy of CAR-NK and CAR-T cells. A recent study by Egli et al. (60) compared autologous CD19-CAR-T cells with allogeneic CD19-CAR-NK cells *in vitro* and *in vivo*. The authors observed stronger antitumor activity with CAR-T cells. Conversely, a report by Marin et al. suggests that allogeneic CAR-NK cells may offer better clinical benefits for specific indolent diseases, such as low-grade non-Hodgkin’s lymphoma (NHL) and chronic lymphocytic leukemia (CLL) (61). These findings highlight the need for further clinical trials to definitively understand the differences in the antitumor efficacy between CAR-NK and CAR-T cells (**Table 3**).

**Table 3:** Comparison of the characteristics of autologous chimeric antigen receptor T (CAR-T) and allogeneic chimeric antigen receptor natural killer (CAR-NK) cell therapies.

	Autologous CAR-T cells	Allogeneic CAR-NK cells
**Antitumor efficacy**	High	High
**Persistence**	Long	Short
**Risk of GvHD**	Low	Low
**Risk of CRS or ICANS**	High	Low
**Time and economic costs**	High	Low
**Off-the-shelf**	Low potential	High potential

GvHD, graft-versus-host disease; CRS, cytokine release syndrome; ICANS, immune effector cell-associated neurotoxicity syndrome.

## 5 Preclinical research on CAR-NK cells

Preclinical studies have yielded promising preliminary data on the efficacy and safety of CAR-NK cell therapy, especially in the context of hematological malignancies. To this end, the following section provides a brief overview of the recent advancements in CAR-NK therapy for the treatment of common hematological tumors.

### 5.1 Leukemia

CD33 is a promising target antigen for CAR-NK cell therapy, being expressed on the surface of leukemia cells in over 90% of patients with acute myeloid leukemia (AML). In the study by Albinger et al. (62), lentiviral transduction was used to generate a CD33-CAR-NK cell model derived from PB-NK cells. These CAR-NK cells demonstrated potent *in vitro* and *in vivo* cytotoxicity against AML cells. Similarly, another study observed a significant reduction in tumor burden within a leukemia mouse model treated with CD33-CAR-PB-NK cells, with no apparent side effects (63). Building on these findings, Zhang et al. engineered CAR-NK cells co-expressing CD33-CAR and anti-CD16 antibodies. This approach achieved dual targeting of AML cells while also enhancing the activity of NK cells (64). Pediatric AML frequently exhibits high CD123 expression, making it another attractive target for CAR-NK therapy. Caruso et al. constructed second-generation CD123-CAR-NK cells utilizing 4–1BB co-stimulatory domains. These CAR-NK cells displayed a superior safety profile compared with CD123-CAR-T cells while maintaining remarkable anti-leukemia activity in both *in vitro* and *in vivo* models (65). The NPM1 mutation is common in AML and has also been targeted using CAR-NK cells. Initial studies suggest promising efficacy with *NPM1*–cytokine-induced memory-like (CIML) CAR-NK cells (66). CD38 represents another potential target for AML immunotherapy. A recent study has demonstrated that the combination of CD38-knockout CAR-NK cells expressing an affinity optimized CD38 with all-*trans* retinoic acid (ATRA) enhanced the targeting ability and the cytotoxicity of NK cells against CD38-positive mature myeloid cells (67). Research on CAR-NK therapy for acute lymphoblastic leukemia (ALL) is also progressing. Studies have explored CD5-CAR-NK cells (68) and CD7-CAR-NK cells (69) for T-cell acute lymphoblastic leukemia (T-ALL) treatment and FLT3-CAR-NK cells in B-cell acute lymphoblastic leukemia (B-ALL) (70). Notably, preclinical experiments using cryopreserved and thawed cord blood CD34^+^ cell-derived CAR-NK cells demonstrated potent and specific antitumor activity against ALL cells (71). The development of dual-targeted CAR-NK cells holds particular promise (72), as evidenced by recent reports of CD19/CD20 dual-targeted CAR-NK cells exhibiting enhanced cytotoxicity against ALL cells (73).

### 5.2 Multiple myeloma

BCMA is a highly expressed antigen on multiple myeloma (MM) cells and represents a promising target for CAR-NK cell therapy (74). CS1, a glycoprotein overexpressed on the MM cell membrane, also offers a potential therapeutic target. CAR-NK cells specifically designed to target CS1 have demonstrated significant antitumor efficacy in preclinical studies (75). GPRC5D, another tumor antigen on MM cells, holds promise for CAR-NK therapy. Yang et al. presented a GPRC5D-targeted CAR-NK product at a recent American Society of Hematology (ASH) meeting (76). This product exhibited potent cytotoxicity, with the efficacy maintained even after cryopreservation and long-distance transport. Dual-targeted CAR-NK cells offer potential advantages over single-targeted BCMA-CAR-NK cells. For instance, BCMA/GPRC5D-CAR-NK cells can effectively lyse BCMA-negative MM cells, leading to improved antitumor efficacy in *in vivo* and *ex vivo* models. This approach has been shown to prolong the survival of mice and to reduce the risk of tumor recurrence (77). FT555, a GPRC5D/CD38-CAR-NK cell product derived from induced pluripotent stem cells (iPSCs), incorporates several features: a GPRC5D-specific CAR structure; a high-affinity, non-cleavable CD16 (hnCD16) domain for enhanced ADCC activity; an IL-15/IL-15 receptor fusion protein (IL-15RF) for sustained cytokine signaling; and CD38 knockout. This product has demonstrated high and persistent killing efficiency, particularly when combined with daratumumab (78). Limited homing of NK cells to tumor sites can hinder the therapeutic efficacy. The co-expression of chemokine receptor 4 (CXCR4) in BCMA-CAR-NK cells has been proposed as a strategy to overcome this limitation, demonstrating promise in preclinical models (79).

### 5.3 Lymphomas

CAR-NK cell therapy has demonstrated continuous progress in the treatment of lymphomas. Gang et al. (80) developed CD19-CAR-CIML-NK cells. These CAR-NK cells exhibited significantly enhanced cytotoxicity against NK-resistant B-cell lymphoma (BCL) cells and improved the overall survival in mouse models. MHC class I chain (MIC)-related proteins act as ligands for NKG2D, effectively activating NK cells. Based on the NKG2D–NKG2DL interaction, Liu et al. designed a unique soluble CAR structure for NK cells named MS-Ig. This molecule comprises a MICA extracellular domain (MICA-ECD) for NKG2D binding, an anti-CD20 single-chain variable fragment (ScFv) for CD20 recognition on tumor cells, and a human IgG Fc fragment. During co-culture with CD20-positive lymphoma cells, the anti-CD20 ScFv binds to CD20 antigens, while MICA-ECD interacts with NKG2D on NK cells, directly triggering the cytotoxicity of NK cells (81). Studies have shown that the combination of anti-CD20 or anti-CD79 antibodies with NKTR-255 (a polymer-conjugated, IL-15Rα-dependent, recombinant human IL-15 agonist) can significantly enhance the *in vitro* cytotoxicity of CD19-CAR-NK cells against Burkitt’s lymphoma (BL) cells (82). CD70, which is predominantly expressed on lymphocytes, has emerged as a potential target for CAR-NK therapy (83). Given the high expression of CD70 on T-cell lymphomas (TCLs), Rafei et al. presented a study at ASH 2023 on a CD70-CAR-NK product for TCL treatment (84). Their findings suggest CD70 as a viable target for CAR-NK therapy against TCLs. Recent reports have further highlighted the excellent efficacy of CD70-CAR-NK cells in CD19-negative BCLs (85).

## 6 Clinical research on CAR-NK cells

Several clinical trials are underway to evaluate the efficacy and safety of CAR-NK cell therapy, with some demonstrating promising initial results. Liu et al. conducted a first-in-human clinical trial of CD19-CAR-UCB-NK cells for CD19-positive CLL or BCL in 11 patients. This trial demonstrated an encouraging overall response (OR) rate of 73% (8/11) and a complete response (CR) rate of 64% (7/11) with no severe side effects, suggesting favorable safety and efficacy (55). The updated follow-up data by Marin et al. (61) showed sustained positive outcomes, with OR rates of 100% for low-grade NHL, 67% for CLL without transformation, and 41% for diffuse large B-cell lymphoma (DLBCL). Notably, high CR rates were observed in indolent diseases (mainly low-grade NHL and CLL). This study also suggests that UCB-NK cells collected within 24 h and with low nucleated red blood cell content (≤8 × 10^7^ cells) may have superior clinical value. Another ongoing clinical trial is evaluating FT596 (CD19-CAR-iPSC-NK cell therapy) for relapsed/refractory (R/R) BCL in 20 patients (86). Early results have indicated an OR rate exceeding 50% (9/17) after the first treatment cycle, with sustained clinical benefit observed in five patients who received a second cycle [four maintained CR and one achieved a deeper partial response (PR)]. The updated data presented at ASH 2021 included 13 patients treated with FT596 monotherapy and 19 treated with FT596 combined with rituximab. The trial also explored different cell doses (30 × 10^6^, 90 × 10^6^, 300 × 10^6^, and 900 × 10^6^) and demonstrated consistent clinical efficacy (NCT04245722). CNTY101, a CD19-CAR-iPSC-NK cell product, achieved its first human dose in February 2023 to assess its safety and efficacy in patients with R/R CD19-positive BCL. A case report presented at the 2023 ASH meeting indicated initial safety and efficacy (NCT05336409). A new PB-CAR-NK cell therapy targeting CD19, NKX019 (NCT05020678), recently reported results at the European Hematology Association (EHA) meeting in a trial involving 19 patients with R/R CD19-positive BCL. In the phase I dose-escalation study, 10 evaluable patients received doses of 1 billion or 1.5 billion CAR-NK cells. The final OR rate was 80% (8/10), with seven patients achieving CR. Importantly, no cases of GvHD or other serious adverse events were observed. While the trial included five patients with ALL/CLL, the therapeutic effect in these patients was less promising. This may be attributed to the limited sample size or the underlying disease mechanisms. Another product from the same company, NKX101 (NKG2D-CAR-NK, NCT04623944), is being evaluated in a clinical trial for AML. The phase I follow-up data presented at ASH 2023 showed a CR/CRi (complete remission with incomplete count recovery) rate as high as 67% (4/6). Additional ongoing clinical trials in AML are also yielding positive outcomes (87). Trials are also underway to assess CAR-NK cell therapy in MM. FT576, a BCMA-CAR-NK cell therapy derived from iPSCs, demonstrated excellent safety and tolerability in patients with R/RMM, both as a monotherapy and in combination with daratumumab. No cases of CRS, neurotoxicity, or GvHD were reported (88). The updated safety data, as of October 7, 2022, and presented at ASH 2022, confirmed no occurrence of serious adverse events (NCT05182073).

A search on ClinicalTrials.gov using the keyword “CAR-NK” identified 44 ongoing clinical trials evaluating CAR-NK cell therapy for hematological malignancies (**Table 4**). The majority of these trials are currently in phase I or II, suggesting that CAR-NK therapy remains in the early stages of clinical investigation.

**Table 4:** Clinical trials of chimeric antigen receptor natural killer (CAR-NK) cell therapy.

NCT no.	Study title	Disease	NK cell source	Target	Phase
NCT05110742	Phase I/II study of CD5 CAR engineered IL15-transduced cord blood-derived NK cells in conjunction with lymphodepleting chemotherapy for the management of relapsed/refractory hematological malignances	T-cell malignances	UCB	CD5	Phase 1/phase 2
NCT05645601	CAR-NK targeted CD19 for R/R B-cell malignancies	R/R B-cell malignancies	PB	CD19	Phase 1
NCT04623944	NKX101, intravenous allogeneic CAR NK cells, in adults with AML or MDS	R/R AML/MDS	PB	NKG2D	Phase 1
NCT06045091	To evaluate the safety and efficacy of human BCMA targeted CAR-NK cells injection for subjects with R/R MM or PCL	R/R MM/plasma cell leukemia	Unknown	BCMA	Early_Phase 1
NCT02742727	CAR-pNK cell immunotherapy in CD7 positive leukemia and lymphoma	CD7-positive leukemia and lymphoma	NK-92	CD7	Phase 1/phase2
NCT05182073	FT576 in subjects with multiple myeloma	MM	iPSC	BCMA	Phase 1
NCT05574608	Allogenic CD123-CAR-NK cells in the treatment of refractory/relapsed acute myeloid leukemia	R/R AML	PB	CD123	Early_Phase 1
NCT06206902	F01 in the treatment of relapsed/refractory non-Hodgkin’s lymphoma	B-cell NHL	Unknown	CD19	Phase 1
NCT06006403	Safety and efficacy of CD123-targeted CAR-NK for relapsed/refractory acute myeloid leukemia or blastic plasmacytoid dendritic cell neoplasm	R/R AML/blastic plasmacytoid dendritic cell neoplasm (BPDCN)	PB	CD123	Phase 1/phase2
NCT03824964	Study of anti-CD19/CD22 CAR NK cells in relapsed and refractory B cell lymphoma	Refractory BCL	Unknown	CD19, CD22	Early_Phase 1
NCT03690310	Study of anti-CD19 CAR NK cells in relapsed and refractory B cell lymphoma	Refractory BCL	Unknown	CD19	Early_Phase 1
NCT05654038	A study of universal CD19-targeted UCAR-NK cells combined with HSCT for B cell hematologic malignancies	B-Cell Lymphoblastic Leukemia/Lymphoma	hPSC	CD19	Phase 1/phase 2
NCT05667155	Clinical study of cord blood-derived CAR NK cells targeting CD19/CD70 in refractory/relapsed B-cell non-Hodgkin lymphoma	B-cell NHL	UCB	CD19, CD70	Phase 1
NCT03692767	Study of anti-CD22 CAR NK cells in relapsed and refractory B cell lymphoma	Refractory BCL	Unknown	CD22	Early_Phase 1
NCT02944162	CAR-pNK cell immunotherapy for relapsed/refractory CD33^+^ AML	R/R CD33^+^ AML	NK-92	CD33	Phase 1/phase 2
NCT04887012	Clinical study of HLA haploidentical CAR-NK cells targeting CD19 in the treatment of refractory/relapsed B-cell NHL	B-cell NHL	Unknown	CD19	Phase 1
NCT05652530	Clinical study of the safety and efficacy of BCMA CAR-NK	R/R MM	Unknown	BCMA	Early_Phase 1
NCT04639739	Anti-CD19 CAR NK cell therapy for R/R non-Hodgkin lymphoma	R/R B-cell NHL	Unknown	CD19	Early_Phase 1
NCT03056339	Umbilical & cord blood (CB) derived CAR-engineered NK cells for B lymphoid malignancies	ALL/CLL/NHL	UCB	CD19	Phase 1/phase 2
NCT05247957	NKG2D CAR-NK cell therapy in patients with relapsed or refractory acute myeloid leukemia	R/R AML	UCB	NKG2D	NA
NCT05739227	Safety and efficacy of allogenic CD19-CAR-NK cells in treatmenting r/r B-cell hematologic malignancies	ALL/BCL/CLL	Unknown	CD19	Early_Phase 1
NCT03579927	CAR.CD19-CD28-zeta-2A-iCasp9-IL15-transduced cord blood NK cells, high-dose chemotherapy, and stem cell transplant in treating participants with B-cell lymphoma	B-cell lymphoma	UCB	CD19	Phase 1/phase 2
NCT05008536	Anti-BCMA CAR-NK cell therapy for the relapsed or refractory multiple myeloma	Refractory MM	UCB	BCMA	Early_Phase 1
NCT05092451	Phase I/II study of CAR.70-engineered IL15-transduced cord blood-derived NK cells in conjunction with lymphodepleting chemotherapy for the management of relapse/refractory hematological malignances	BCL/MDS/AML	UCB	CD70	Phase 1/phase 2
NCT05487651	Allogeneic NK T-cells expressing CD19 specific CAR in B-cell malignancies	NHL/BCL/DLBCL/B-cell leukemia	PB	CD19	Phase 1
NCT03940833	Clinical research of adoptive BCMA CAR-NK cells on relapse/refractory MM	MM	NK-92	BCMA	Phase 1/phase 2
NCT05570188	Anti-CD19 universal CAR-NK cells therapy combined with HSCT for B cell hematologic malignancies	BCL/B-cell leukemia	Unknown	CD19	Phase 1/phase 2
NCT06325748	SENTI-202: Off-the-shelf logic gated CAR NK cell therapy in adults with CD33 and/or FLT3 blood cancers including AML/MDS	CD33 and/or FLT3 AML/MDS	PB	CD33/FLT3	Phase 1
NCT05472558	Clinical study of cord blood-derived CAR-NK cells targeting CD19 in the treatment of refractory/relapsed B-cell NHL	R/R B-cell NHL	UCB	CD19	Phase 1
NCT05008575	Anti-CD33 CAR NK cells in the treatment of relapsed/refractory acute myeloid leukemia	R/R AML	UCB	CD33	Phase 1
NCT04796675	Cord blood derived anti-CD19 CAR-engineered NK cells for B lymphoid malignancies	ALL/CLL/NHL	UCB	CD19	Phase 1
NCT05410041	Anti-CD19 CAR-engineered NK cells in the treatment of relapsed/refractory B-cell malignancies	B-cell malignances	Unknown	CD19	Phase 1
NCT06242249	Anti-BCMA CAR-NK therapy in relapsed or refractory multiple myeloma	R/R MM	Unknown	BCMA	Phase 1/phase 2
NCT05842707	Study of cord blood-derived CAR NK cells targeting CD19/CD70 in refractory/relapsed B-cell non-Hodgkin lymphoma	R/R B-cell NHL	UCB	CD19, CD70	Phase 1/phase 2
NCT05020015	A study of TAK-007 in adults with relapsed or refractory (r/r) B-cell non-Hodgkin lymphoma (NHL)	R/R B-cell NHL	UCB	CD19	Phase 2
NCT05336409	A study of CNTY-101 in participants with CD19-positive B-cell malignancies	Indolent B-cell NHL/aggressive B-cell NHL	iPSC	CD19	Phase 1
NCT05020678	NKX019, intravenous allogeneic chimeric antigen receptor natural killer cells (CAR NK), in adults with B-cell cancers	B-cell malignancies	PB	CD19	Phase 1
NCT04747093	Induced-T cell like NK cells for B cell malignancies	B-cell malignancies	Unknown	CD19	Phase 1/phase 2
NCT05563545	Anti-CD19 CAR-engineered NK cells in the treatment of relapsed/refractory acute lymphoblastic leukemia	R/R ALL	Unknown	CD19	Phase 1
NCT06201247	Off-the-shelf CD123 CAR-NK for R/R AML	R/R AML	PB	CD123	Early_Phase 1
NCT06307054	CLL-1 CAR-NK cells for relapsed/refractory AML	R/R AML	Unknown	CLL-1	Phase 1
NCT05734898	NKG2D CAR-NK & r/rAML	R/R AML	Unknown	NKG2D	NA
NCT06027853	Natural killer (NK) cell therapy targeting CLL1 in acute myeloid leukemia	AML	iPSC	CLL1	Phase 1
NCT05673447	The study of anti-CD19 CAR NK cells in the treatment of relapsed/refractory diffuse large B cell lymphoma	DLBCL	Unknown	CD19	Early_Phase 1

R/R, relapsed/refractory; MDS, myelodysplastic syndromes; CLL, chronic lymphocytic leukemia; MM, multiple myeloma; NHL, non-Hodgkin’s lymphoma; ALL, acute lymphoblastic leukemia; AML, acute myeloid leukemia; DLBCL, diffuse large B-cell lymphoma; UCB, umbilical cord blood; PB, peripheral blood; iPSC, induced pluripotent stem cell; hPSC, human pluripotent stem cell; BCMA, B-cell maturation antigen.

## 7 Current challenges

Despite the promising preclinical and clinical data on the efficacy and safety of CAR-NK cell therapy for hematological malignancies, several limitations need to be addressed before its widespread clinical application. These limitations are summarized in **Table 5**.

**Table 5:** Limitations of and potential solutions for chimeric antigen receptor natural killer (CAR-NK) cell therapy.

Limitations	Solutions	References
**Unsatisfactory targeted antitumor efficacy**	Dual- or multi-specific target CARs Specially modified NK cells CARs with different affinity Suicide switches	(89, 90) (91) (57) (55, 92, 93)
**Short persistence of CAR-NK cells**	Lymphodepleting conditioning Multiple infusions of CAR-NK cells Exogenous cytokine support Application of CIML Design of AI-CARs	(94) (95) (32, 55, 96, 97) (66, 98) (99, 100)
**Inhibition of the tumor microenvironment**	Targeting at components of the TME Manipulating the expansion conditions Modifying CAR-NK cells Targeting at specific inhibitory checkpoint	(101) (102) (103–105) (106–109)

CIML, cytokine-induced memory-like; AI-CARs, CARs expressing both activating and inhibitory CARs; TME, tumor microenvironment.

### 7.1 Improving the targeted antitumor efficacy

Ideally, the CAR targets should exhibit high specificity, a broad coverage of tumor cells, and stable expression on malignant cells. However, achieving all these characteristics simultaneously is challenging, and few targets in clinical application fully meet these criteria (110). To enhance the antitumor efficacy, the development of dual- or multi-specific CARs has emerged as a promising strategy (111). A clinical trial using CD19/CD22-CAR-T cells for refractory ALL has demonstrated the potential of dual-CARs. All patients achieved complete remission with no minimal residual disease (MRD), highlighting their clinical efficacy (89). Similarly, bispecific CAR-NK cells targeting both CD19 and BCMA for B-ALL and MM have shown potent cytotoxicity *in vitro*, warranting further investigation of their *in vivo* efficacy (90). Moreover, triple-modified NK cells incorporating a CD19-targeting CAR, hnCD16, and an IL-15/IL-15 receptor fusion protein (IL-15RF) have been designed, potentially mitigating tumor escape to a greater extent (91). Ensuring safety is paramount in CAR-NK cell therapy. The CAR design should not only accurately target tumors but also minimize the risk of “on-target, off-tumor” toxicity against normal tissues expressing the target antigen. Studies in various tumor models have demonstrated the feasibility of utilizing CARs with lower affinity for tumor tissues expressing high levels of TAA. This approach ensures that only those tumor cells with a sufficiently high antigen density can activate the CAR structure (57). Furthermore, incorporating suicide switches into CAR-NK cells offers an additional safety measure (92). The inducible caspase 9 (iCasp9) system has been successfully employed for this purpose, demonstrating a favorable safety profile (55, 93).

### 7.2 Extending the persistence of CAR-NK cells

Unlike T cells, which can differentiate into memory cells and maintain long-term antitumor efficacy (110), NK cells have a shorter life span in humans (approximately 2 weeks in blood) (112). This limited life span offers some safety advantages, but also hinders the efficacy of CAR-NK cell therapy (103). Various strategies have been implemented to prolong the survival of NK cells *in vivo*. Lymphodepleting conditioning regimens prior to adoptive cell transfer can mitigate the risk of rejection and significantly improve the efficacy of cell therapy through various mechanisms (94). Therefore, the combination of cyclophosphamide and fludarabine is often used as pretreatment before clinical treatment with allogeneic CAR-NK cells, whereas autologous NK cells do not necessitate pretreatment (113). Multiple infusions of CAR-NK cells have also been proposed to potentially safeguard the treatment outcomes (95). However, the inherent heterogeneity of the natural immune system requires close monitoring (114) and assessment of host immune rejection against allogeneic NK cells to prevent diminished efficacy (115, 116). Exogenous cytokine support, particularly IL-2 and IL-15, can maintain the proliferation and activity of NK cells *in vivo* (97, 117). However, systemic high-dose injections could induce additional safety risks, as exemplified by the association between high-dose IL-2 therapy and vascular leak syndrome (118). Liu et al. (32, 55) addressed these limitations by constructing CD19-CAR (iC9/CAR.19/IL-15)-CB-NK cells that ectopically secrete IL-15 and express suicide switches. These cells exhibited both longer persistence and stronger antitumor efficacy *in vitro* and *in vivo*, persisting at low levels for at least 12 months in patients. Building on this concept, Daher et al. (96) constructed *CISH*-knockout (KO)/IL-15 CAR-NK cells using CRISPR-Cas9 to knock down IL-15-associated negative immune checkpoint genes (*CISH* genes). These *CISH*-KO/IL-15 CAR-NK cells exhibited enhanced survival and antitumor activity compared with the control IL-15 CAR-NK cells. The induction of immune memory in NK cells represents another approach to improve their activity (119). NK cells can acquire memory-like properties upon stimulation with cytokines, particularly IL-12, IL-15, and IL-18. Therefore, the application of cytokines to induce the generation of memory-like NK cells might prolong their *in vivo* survival (66, 98). Interestingly, some studies have suggested that activating CAR (aCAR) NK cells can express and acquire trogocytic antigens (TROGs) through trogocytosis, leading to fratricide (killing of other CAR-NK cells). However, co-expressing an inhibitory CAR (iCAR) on these aCAR-NK cells can restrain their killing function, thereby reducing the risk of fratricide. This phenomenon suggests that CARs expressing both aCAR and iCAR (AI-CARs) could not only minimize NK cell depletion but also improve the durability of CAR-NK cell therapy *in vivo* (99, 100).

### 7.3 Overcoming the inhibitions of the tumor microenvironment

The tumor microenvironment (TME) presents a significant challenge for effective immune cell killing. Cancer-associated fibroblasts (CAFs), a major component of the tumor stroma, are well-established contributors to tumor progression (120). The targeting of CAFs represents a potential strategy to counteract the negative influence of the TME. Sakemura et al. (101) explored this concept by designing BCMA/CAF-CAR-T cells. Studies using human MM cells and mouse models demonstrated that this dual-targeted CAR structure could reverse CAR-T cell dysfunction and enhance the antitumor efficacy. In addition, manipulation of the expansion conditions of CAR-NK cells might mitigate the suppressive effects of the TME, as has been tentatively demonstrated with CAR-T cells (102). The TME harbors multiple immunosuppressive factors, including hypoxia, transforming growth factor beta (TGF-β), PGE-2, and extracellular metabolites such as lactate and adenosine, which can restrain immune cell activity (121). The modification of NK cells to overcome these suppressive factors presents a promising approach. Knockdown of the TGF-β receptor II (TGF-βRII) on NK cells has been shown to partially overcome the negative influence of the TME without compromising their anti-leukemia efficacy (103). Engineering CARs that express negative TGF-β receptors and combining treatment with TGF-β receptor inhibitors are also being explored (104, 105). Tumor cells can also upregulate the expression of ligands for immune cell inhibitory receptors, exploiting the negative feedback mechanisms of the immune system to evade immune clearance. Strategies to address this challenge include the knockdown of relevant receptors on NK cells (such as NKG2A) or combining therapy with specific inhibitory checkpoint antibodies to improve the antitumor effects (106–108). Furthermore, the design of CAR structures that target inhibitory checkpoints represents a promising approach (109), as initially demonstrated in solid tumors (122).

## 8 Conclusions

NK cells offer a novel avenue for cancer immunotherapy, and the development of CAR-NK cell therapy holds significant promise for the treatment of hematological malignancies. Preclinical studies suggest that CAR-NK cell therapy might address some of the limitations associated with CAR-T cell therapy while achieving comparable clinical efficacy. While NK cells and CAR-NK immunotherapy have inherent limitations, researchers are actively developing strategies to improve their treatment efficacy. Early studies have yielded promising results, paving the way for future clinical applications of CAR-NK therapy.

## Author contributions

RY: Writing – original draft, Writing – review & editing. YY: Supervision, Writing – review & editing. RL: Writing – review & editing. YW: Writing – review & editing. RYY: Writing – review & editing. AH: Conceptualization, Supervision, Writing – review & editing.
